# Comparative effects of bone marrow stimulation and shockwave therapy on rotator cuff healing in chronic massive rotator cuff tears: A rat model study

**DOI:** 10.1002/jeo2.70622

**Published:** 2026-01-11

**Authors:** Utku Demirtaş, Elif Mercan Demirtaş, Mert Özcan, Fulya Öz Puyan

**Affiliations:** ^1^ Department of Orthopedics and Traumatology Sultan 1. Murat State Hospital Edirne Turkey; ^2^ Department of Pathology Trakya University School of Medicine Edirne Turkey; ^3^ Department of Orthopedics and Traumatology Trakya University School of Medicine Edirne Turkey

**Keywords:** biological augmentation, bone marrow stimulation, chronic massive rotator cuff tear, extracorporeal shockwave therapy (ESWT), tendon‐to‐bone healing

## Abstract

**Purpose:**

Surgical intervention for massive rotator cuff tear (MRCT) has been found to result in positive outcomes; however, a high retear rate has frequently been reported. The present study employed a chronic MRCT model to assess the impact of microdrilling, a bone marrow stimulation (BMS) technique and extracorporeal shockwave therapy (ESWT) on rotator cuff healing. We hypothesized that BMS would result in superior healing than ESWT, reflected by a more normal tendon‐to‐bone junction histomorphology.

**Methods:**

A chronic rotator cuff tear model was created in the supraspinatus and infraspinatus tendons of 30 Sprague–Dawley rats. Four weeks later, the tendons were repaired using the transosseous technique. The animals were randomly assigned to three groups. In Group I, the intervention consisted exclusively of tendon repair. In Group II, ESWT was administered at various time points in conjunction with the repair process. In Group III, microdrilling was performed prior to the initiation of repairs. The animals were euthanized at 4 and 8 weeks post‐repair. Repaired tendons, specifically the tendon‐to‐bone junction, were evaluated histomorphologically using the Bonar score and immunohistochemically for CD34, bone morphogenetic protein‐2 (BMP‐2), bone morphogenetic protein‐7 (BMP‐7) and transforming growth factor‐β1 (TGF‐β1).

**Results:**

A lower Bonar scale score in Group III more closely resembled normal tissue with respect to tenocyte morphology, extracellular matrix composition and vascularity, although statistical significance was limited to vascularity and extracellular matrix composition. By contrast, Group II showed a significant time‐dependent deterioration in tenocyte morphology. Group III exhibited elevated BMP‐2 and BMP‐7 expression levels, and an increase in CD34 at the tendon‐to‐bone junction was observed in Group II; however, these did not reach statistical significance. TGF‐β1 expression remained comparable among all groups.

**Conclusions:**

Both biological augmentation with BMS and ESWT demonstrated beneficial effects on tendon‐to‐bone healing in a rat model of chronic MRCT; however, these effects were not statistically significant. Although increased BMP‐2, BMP‐7 and CD34 expressions were noted in Group III, significant improvements were noted in vascularity according to the Bonar scale score, along with early enhancements in ground substance and overall histological structure. The deterioration of tenocyte morphology in the ESWT group highlights the need for optimized energy parameters.

**Level of Evidence:**

N/A.

**Abbreviations**
BMP‐2: bone morphogenetic protein‐2
BMP‐7: bone morphogenetic protein‐7
BMS: bone marrow stimulation
ECM: extracellular matrix
ESWT: extracorporeal shockwave therapy
H&E: hematoxylin–eosin
HS: humerus shaft
IS: infraspinatus tendon
MRCT: massive rotator cuff tear
MSCs: mesenchymal stem cells
RC: rotator cuff
RCT: rotator cuff tear
SS: supraspinatus tendon
TGF‐β1: transforming growth factor‐β1
TO: transosseous tunnel

## INTRODUCTION

Rotator cuff tears (RCTs) are a prevalent cause of shoulder pain, affecting millions of individuals worldwide. Massive rotator cuff tears (MRCT) account for approximately 10%–40% of all RCTs [9]. Despite the absence of a universally accepted definition, MRCTs are most frequently characterized as tears greater than 5 cm in diameter or as complete detachment of two or more tendons from the proximal humerus [14].

Surgical repair remains the primary treatment option for MRCTs; however, retear rates remain high, ranging from 20% to 94% [14, 28]. Factors such as advanced age, tendon retraction, fatty degeneration, scarring and poor tissue quality contribute to the technical difficulty of repair and increase the likelihood of failure [10]. Furthermore, MRCTs are distinguished by disrupted collagen alignment, diminished cellularity and inadequate vascularity, all of which negatively impact the biological healing process [20].

Recent studies have centred on biological augmentation strategies to enhance healing [22, 27, 34]. Bone marrow stimulation (BMS) has garnered attention due to its simplicity, safety and inexpensive and intraoperative feasibility, which represents a potentially cost‐effective biological approach to enhancing healing. These include the application of exosomes, growth factors, mesenchymal stem cells (MSCs), scaffolds or combinations of these. MSCs derived from bone marrow (BM) have demonstrated potential in promoting tendon‐to‐bone healing [17].

BMS, a procedure routinely employed for cartilage repair in the knee and ankle, has been adapted for utilization in RC repair [2]. It is hypothesized that the presence of microperforations in the greater tuberosity during surgical procedures enables the release of BM‐derived cells and growth factors, thereby promoting enhanced local healing environments [16]. However, the clinical evidence regarding its efficacy in MRCT repair remains inconclusive [18].

Extracorporeal shockwave therapy (ESWT), a non‐invasive treatment that applies high‐amplitude acoustic waves to stimulate tissue healing and preliminary investigations indicate that ESWT may encourage the formation of new blood vessels, enhance tissue and nerve regeneration, have chondroprotective, anti‐inflammatory and anti‐apoptotic effects and promote enhanced tendon‐to‐bone healing [8, 23].

These biological augmentations have been proposed to enhance healing in the literature, yet direct comparison data within a single chronic MRCT model remain limited. Therefore, we designed a massive chronic rat study to compare BMS and ESWT within the same experimental framework. We hypothesized that BMS would be more effective than ESWT, yielding a more normal enthesis histomorphology (lower Bonar scores) and thereby superior tendon‐to‐bone healing.

## MATERIAL AND METHODS

The present study was conducted on a group of 30 adult male Sprague–Dawley rats, all of which were derived from a single breeding pair and aged 14 weeks and weighed between 450 and 530 g. The study protocol was approved by the Trakya University Local Ethics Committee for Animal Experiments (date: 27 December 2019/12). The rats were maintained under identical conditions and fed a consistent diet. All rats were housed (five per housing unit) under controlled conditions, including a regulated temperature and humidity, and maintained on a consistent 12:12 h light–dark cycle in accordance with their nocturnal behaviour. The subjects were provided with food and water ad libitum, without any dietary restrictions.

All surgical interventions were performed on the right shoulder. Each animal underwent two surgical procedures. The first procedure induced a chronic, massive, retracted rotator cuff (RC) tear, and the second procedure, performed 4 weeks later, involved tendon repair. In this study, our primary objective was to directly compare two augmentation techniques (BMS and ESWT) within the context of chronic surgical repair. For this reason, the group that underwent repair only (Group I) was intentionally designated as the reference (control) group, representing the standard surgical treatment without biological enhancement. This design allowed a direct evaluation of the two augmentation methods against a histomorphologically and immunohistochemically relevant baseline while adhering to the small sample size.

Despite the evident disparities in rat shoulder anatomy and biomechanics when compared with those observed in humans, the Sprague–Dawley rat is regarded as a generally accepted and extensively utilized model for the study of RC pathology and repair in the context of preclinical research [4, 38]. To represent the transosseous tunnel (TO) part of the procedure, a generic three‐dimensional (3D) model of the humerus (STL file) was downloaded from the repository associated with the study [3]. The model was then rescaled according to the morphometric measurements of the Sprague–Dawley rat humerus reported and 3D‐printed for use in the demonstration of the procedure [5].

## INTERVENTION

All surgical procedures were performed under aseptic conditions and general anaesthesia using intramuscular injections of 50 mg/kg ketamine HCl (Ketalar®; Pfizer) and 5 mg/kg xylazine HCl (Xylazin Bio 2%®; Bioveta). Under aseptic conditions, a 2 cm skin incision was made over the superolateral right shoulder. Subcutaneous tissue and fat were dissected to expose the deltoid, which was opened with an inverted T; the proximal anterior, lateral and posterior fibres were detached from the acromion and clavicle. The supraspinatus tendon (SS) and infraspinatus tendon (IS) were identified. The SS and IS tendons were detached in tendon‐to‐bone junction (enthesis) area, tagged proximally with 3‐0 silk sutures and transected perpendicular to its long axis ~4 mm proximal to its insertion, allowing proximal retraction and exposure of the humeral head cartilage. Care was taken to preserve the remaining cuff tendons and glenohumeral structures. Muscle, subcutaneous tissue and skin were closed with 3‐0 Prolene (Ethicon). Subsequent to the surgical procedure, the subjects were returned to their cages without limb immobilization, and full weight‐bearing was permitted as tolerated, which further minimized the likelihood of spontaneous healing at the enthesis area.

**Figure 1:**
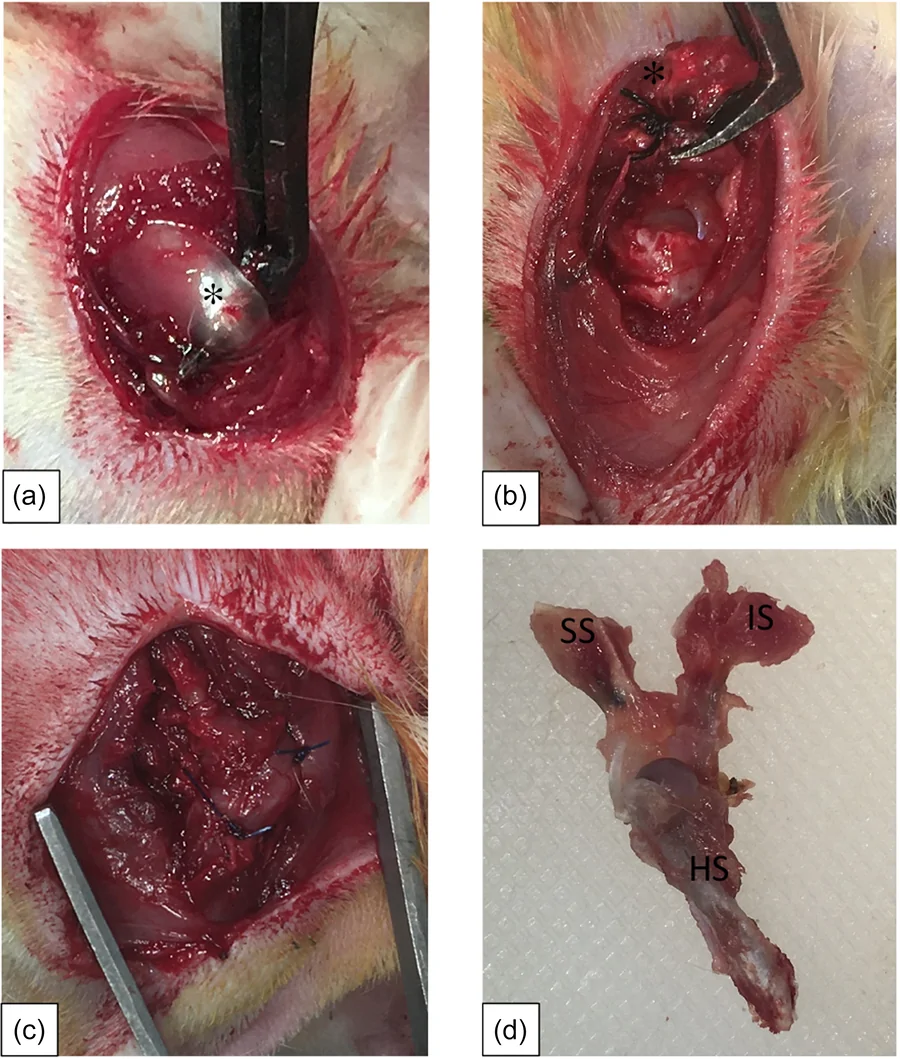
Macroscopic intraoperative views of sequential surgical steps performed in the study. (a) SS and IS tendons were identified and isolated prior to detachment from their insertion sites. (b) SS and IS tendons, previously marked with 3‐0 silk sutures during the first surgery, are shown prior to tendon repair. The sutures allowed accurate identification and mobilization of the retracted tendon stumps before reattachment. (c) SS and IS tendons were reattached to their anatomical footprint on the proximal humerus using a single‐row suture configuration. The repair site is shown after fixation was completed. (d) Macroscopic view of the resected tendon‐to‐bone complex prior to pathological examination. The specimen includes the SS and IS tendons along with the humerus shaft (HS), harvested as a single unit for histopathological and immunohistochemical analysis. IS, infraspinatus tendon; SS, supraspinatus tendon.

Following a 4‐week period, all animals underwent a second surgical procedure to simulate a chronic RC tear repair model. The 4‐week delay between tendon detachment and repair, followed by evaluations at 4 and 8 weeks post‐repair, was selected to model chronic degenerative changes. This protocol remains an accepted and validated model for chronic RCTs in rats, as demonstrated in recent studies [19, 26, 36]. The initial incision was then reopened, and the silk markers were meticulously located and removed. At this juncture, the procedures diverged based on the subject's group affiliation (Figure 1).

**Figure 2:**
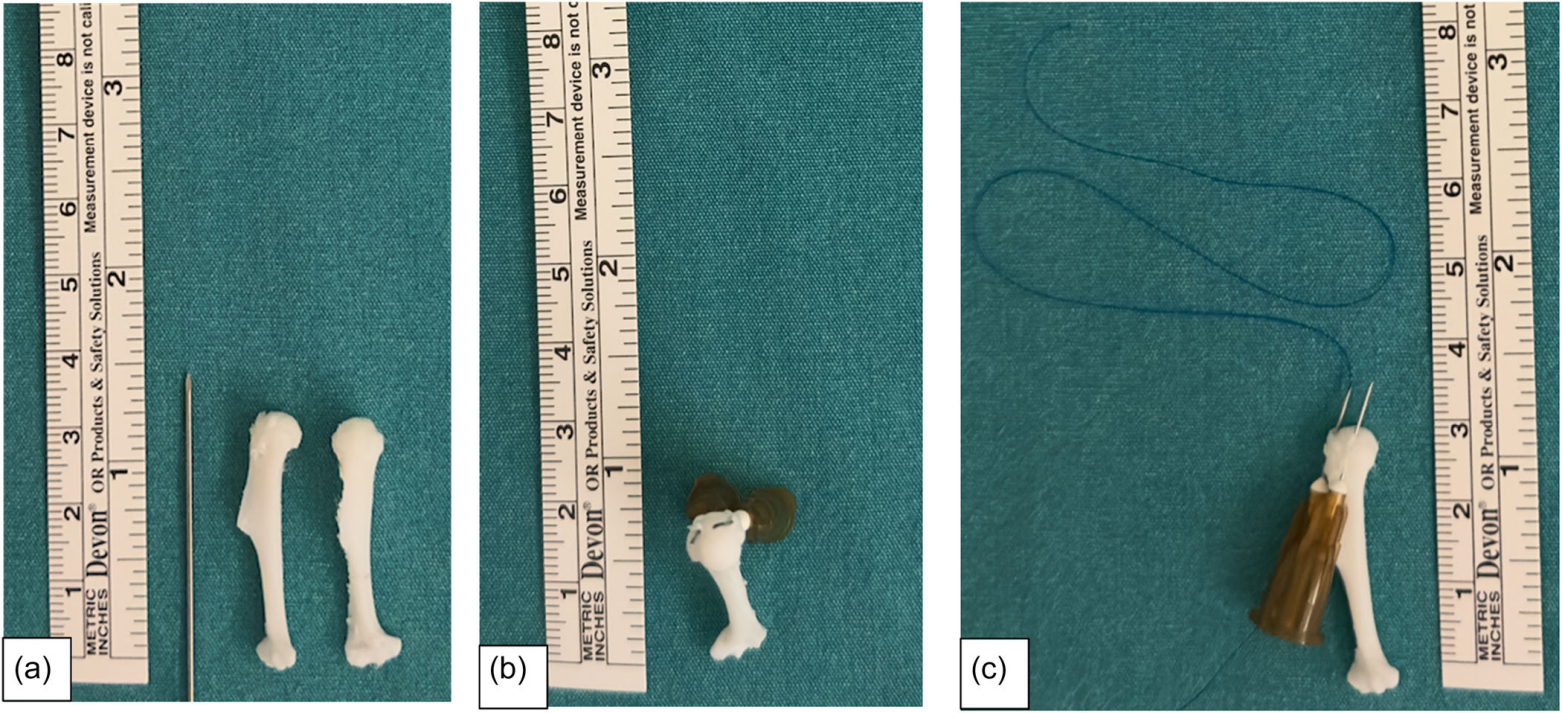
Demonstration of the humeral fixation was achieved via a transosseous tunnel (TO) performed on a 3D‐printed rat humerus model. (a) Two TO drilled in the greater tuberosity with a 1.0‐mm Kirschner wire. (b) Superior view of the 3D‐printed model with two subcutaneous needles passed through the SS and IS tendon footprint to simulate a transosseous suture technique. (c) Suture was shuttled through the tunnel to reduce using a subcutaneous needle on the IS and SS to its footprint. 3D, three‐dimensional; IS, infraspinatus tendon; SS, supraspinatus tendon.

Group I, Control Group and Group II, ESWT Group, the SS and IS tendons were sutured using a modified Masson‐Allen technique with 3‐0 Prolene sutures. Its humeral fixation was achieved via a TO drilled in the greater tuberosity with a 1.0‐mm Kirschner wire. Using a subcutaneous needle, the tendon suture was shuttled through the tunnel to reduce the IS and SS to its footprint [26] (Figure 2). In order to prevent interference with the healing process between the tendon and the bone, knots were strategically positioned at a distance from the site of the repair. Muscle, subcutaneous tissue and skin were closed with 3‐0 Prolene (Ethicon).

Group III, the BMS group, prior to the initiation of the repair procedure, the BMS was performed by means of drilling two 1‐mm holes into the medialized microdrilling to the footprint area using a surgical drill manufactured by Shanghai Bojin Medical Instrument. Kirschner wires were inserted through the cortex into the cancellous bone to access the BM [16]. The repair of the tendons was then executed using the same technique as in Groups I and II.

Subsequent to the surgical procedure, the rats were returned to their cages without the imposition of activity restrictions. Two weeks after the completion of the repair, rats in Group II received ESWT under anaesthesia according to low energy parameters (approximately 0.20 mJ/mm^2^). ESWT was administered via the Masterpuls® MP50 device (Storz Medical AG), with the application of gel to the subject's shoulder and the delivery of 1000 impulses at an intensity of 1.0 bar and a frequency of 10 Hz.

At 4 weeks post‐repair, five rats from each group were randomly selected for sacrifice. The established anaesthesia and cervical dislocation protocol was used to euthanize the animals. The right proximal humerus, encompassing the tendon–bone interface and the attached SS and IS muscles, was harvested for subsequent histological and immunohistochemical analysis. Samples were preserved in 10% formaldehyde in labelled, sterile 100‐mL containers (Fratmed).

At 6 weeks post‐repair, the five remaining rats in Group II underwent a second round of ESWT using the same protocol. At 8 weeks post‐repair, the remaining animals were euthanized, and samples were collected for further analysis.

The 4‐week time point has consistently been recognized as the stage at which the tendon injury exhibits chronic degenerative features, including fibroblast proliferation, neovascularization and collagen disorganization. These findings closely parallel chronic human rotator cuff pathology. The 8‐week post‐repair period, on the other hand, generally represents a phase of active tissue remodelling and functional recovery, characterized by better collagen alignment, increased fibrocartilage formation and enhanced biomechanical strength at the tendon–bone interface [4, 26]. Therefore, selecting these two time points allowed us to evaluate both the establishment of chronic degeneration and the subsequent remodelling and healing response.

## OUTCOME MEASUREMENTS

### Preparation of samples for evaluation

Following fixation in 10% formalin for 24 h, all samples were decalcified in 10% formic acid until they were suitable for sectioning. To this end, longitudinal sections were obtained to visualize the bone–tendon interface and subsequently processed using the Tissue‐Tek VIP® 6 AI automatic tissue processor (Sakura®). Subsequent to a 12‐h processing cycle, the samples were embedded in paraffin. Subsequently, sections with a thickness of 4 μm were obtained by cutting the paraffin blocks.

For the purpose of histopathological evaluation, hematoxylin–eosin (H&E) staining and Masson's trichrome staining were performed in accordance with standard protocols, and for immunohistochemical analysis, antibodies against bone morphogenetic protein‐2 (BMP‐2), bone morphogenetic protein‐7 (BMP‐7) and transforming growth factor‐β1 (TGF‐β1) were obtained from Invitrogen (Thermo Fisher Scientific) and CD34 from Dako (Glostrup). Paraffin‐embedded sections (4 μm) were mounted on slides and stained using the Ventana Benchmark XT/ISH automated system (Roche) and the OptiView Universal DAB Detection Kit. The following optimization process was employed to determine the optimal incubation times for antibodies: The duration for BMP‐2 was 52 min, for BMP‐7 40 min, for TGF‐β1 32 min and for CD34 56 min. For each rat, four H&E‐stained sections were obtained to evaluate overall tissue morphology. In addition, one representative section per animal was used for each of the stainings and histomorphology analyses and immunohistochemical analyses.

A thorough examination of all prepared slides was conducted under a light microscope (Eclipse Ci‐L; Nikon). The evaluations were centred on the tendon‐bone interface, the presence of inflammation, fibrosis and a foreign body reaction.

### Gross evaluation

At the time of tissue harvesting, a macroscopic evaluation of all specimens was conducted to assess the extent of tendon healing at the tendon–bone interface. The degree of healing was determined through a comprehensive evaluation that included a thorough visual inspection of tendon continuity, the integrity of reattachment to the bone and the presence or absence of scar tissue.

### Histopathological evaluation

Slides that had been stained with H&E and Masson's trichrome were evaluated by a senior pathology resident under the supervision of an experienced pathologist. All evaluations were conducted in a blinded manner, with the evaluators unaware of the group assignments. In an effort to minimize bias and enhance reliability, the assessments were administered at two distinct time points. The histological evaluation was conducted using the Bonar scoring system, a methodical approach that ranges from 1 to 3, and encompasses the assessment of tenocyte morphology, the composition of the extracellular matrix (ECM), the organization of collagen fibres and the vascularity present in the tissue. A low score indicates that the tissue structure is within normal limits or is close to normal, suggesting the absence or minimal presence of degenerative changes or pathological alterations.

### Immunohistochemical evaluation

Immunohistochemically stained sections were meticulously examined by an experienced pathologist and a senior pathology resident, who were both unaware of the study groups. Evaluations were performed at two separate time points, similar to the histological analysis. The expression levels of BMP‐2, BMP‐7 and TGF‐β1 were evaluated using a semi‐quantitative immunohistochemical scoring system [37]. These markers were chosen to represent the key biological processes involved in tendon‐to‐bone healing. In rotator cuff pathology, excessive mechanical load and injury lead to downregulation of TGF‐β1 expression in the tendon. Macrophages at the injury site secrete TGF‐β1 during the healing phase, which stimulates collagen synthesis and matrix remodelling. Its evaluation, therefore, reflects the level of reparative fibrogenic activity within the tendon [13]. The BMP family, a subset of the TGF‐β superfamily, plays a crucial role in the regeneration of the enthesis through promotion of fibrocartilage and bony ingrowth. BMP‐2 and BMP‐7 have demonstrated strong osteoinductive potential. Their expression indicates osteogenic differentiation and remodelling at the repair site [39].

CD34 is an adhesion molecule that is found on the surface of two types of cells: hematopoietic stem cells and vascular endothelial cells. Microvascular density was quantified by identifying the three areas of highest immunoreactivity within each section. In each area, positively stained microvessels were enumerated at ×200 magnification, and the mean value was calculated to determine the microvessel density [25].

All histomorphological and immunohistochemical evaluations, including CD34 quantification, were performed specifically at the tendon‐to‐bone interface, which was identified based on consistent anatomical landmarks in each specimen. The comparison across groups was restricted to the tendon–bone junction, avoiding the potential bias that could arise from evaluating different tissue zones. Fatty infiltration was not evaluated and is therefore not reported in the study.

### Statistical analysis

Statistical analyses were conducted using IBM SPSS Statistics for Windows (version 16.0; IBM Corp.). Continuous variables, Bonar scale score and CD34 were summarized as mean ± standard deviation (SD) according to distribution; categorical variables, BMP‐2, BMP‐7 and TGF‐β, as percentages. Normality was evaluated with the Shapiro–Wilk test and homogeneity of variances with Levene's test. For quantitative variables comparisons of Bonar scale score and CD34, Student's *t* test and one‐way analysis of variance were used for approximately normal data; otherwise, Kruskal–Wallis tests were applied for three‐group comparisons with pairwise Mann–Whitney *U* tests as post hoc. Categorical variables, BMP‐2, BMP‐7 and TGF‐β, were compared using the Pearson *χ* ^2^ test (or Fisher's exact test when expected counts were <5). All tests were two‐sided; a *p* value < 0.05 was considered statistically significant, and 95% confidence intervals (95% CIs) were reported. Inter‐rater reliability based on two readings per specimen was evaluated using the intraclass correlation coefficient (ICC) with a two‐way random‐effects, absolute‐agreement, average‐measures model (ICC[2,2]), and 95% CIs were reported. Post‐hoc power values performed. Primary analysis Bonar scores were compared between Group II and Group III at all time points.

## RESULTS

### Gross evaluation

Macroscopic examination revealed that all specimens exhibited retraction at the tendon–bone junction during the repair process. The tendon stumps exhibited adhesion to the surrounding soft tissues and were enveloped in scar tissue at the insertion site. At the time of euthanasia, both the SS and IS tendons were observed to be attached to their respective footprints on the greater tuberosity, with no visible defects at the tendon‐to‐bone interface in any of the samples.

### Histopathological evaluation

Across all study groups, degeneration of tenocyte morphology was associated with enlarged, rounded nuclei, expansion of the cytoplasm and the formation of lacunae. In samples where the ECM appeared normal, no staining of the ground substance was observed. However, in regions exhibiting abnormal characteristics, a buildup of mucin between collagen fibrils was observed, along with a reduction in the distinction of fibril bundles. The disruption of collagen structure progressed from the blurring of bundle boundaries and fibrillar separation to the complete loss of organized collagen architecture.

**Figure 3:**
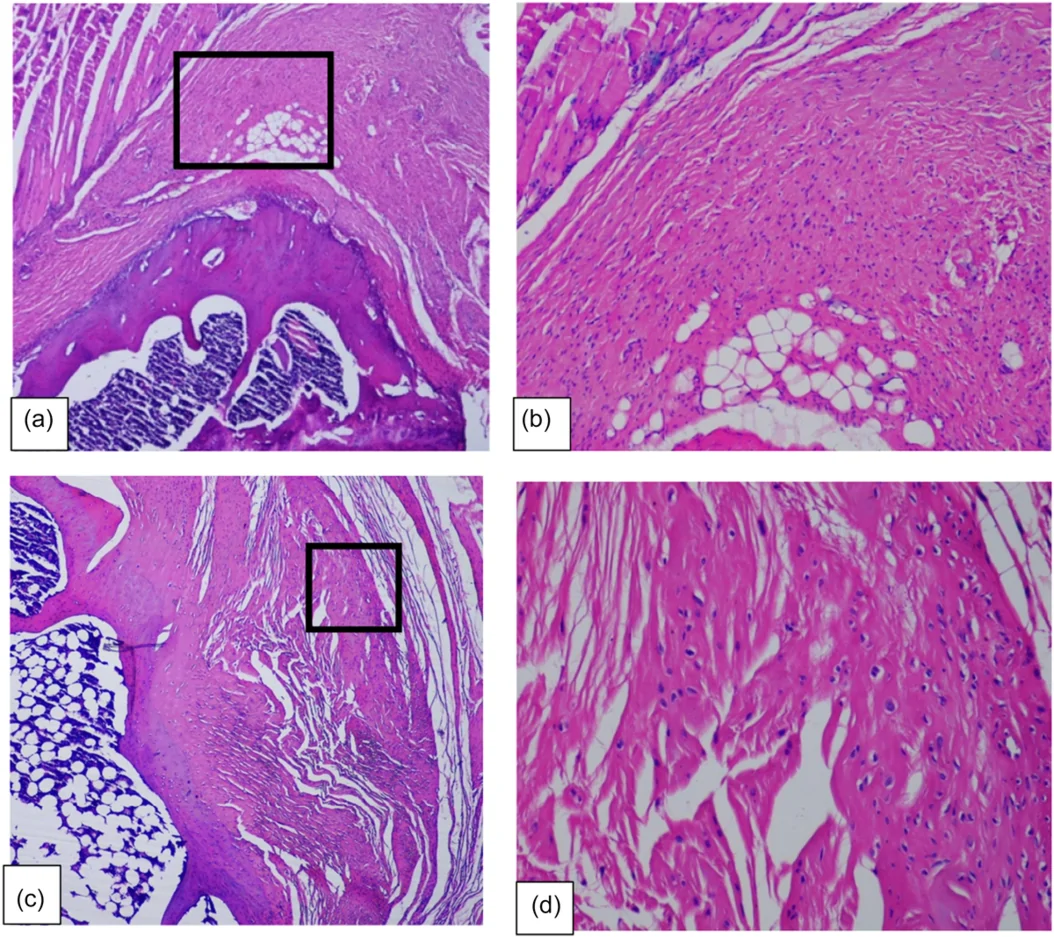
Histologic evaluation of tendon degeneration in the Group II (H&E staining). (a) Dense collagenous tendon tissue showing focal adipocytic metaplasia and fibrocartilaginous change at the enthesis. Adjacent skeletal muscle fibres are noted (H&E staining, ×40 magnification). (b) Black‐boxed area in (a) is shown at higher magnification in (b). Tenocytes appear elongated with reduced cytoplasmic volume and focal adipocytic metaplasia (H&E staining, ×100 magnification). (c) Disorganized and wavy collagen fibres within the tendon (H&E staining, ×40 magnification). (d) Black‐boxed area in (c) is shown at higher magnification in (d). Tenocytes show round, centrally located nuclei and a more conspicuous cytoplasm within expanded lacunar structures (H&E staining, ×100 magnification). H&E, hematoxylin–eosin.

**Figure 4:**
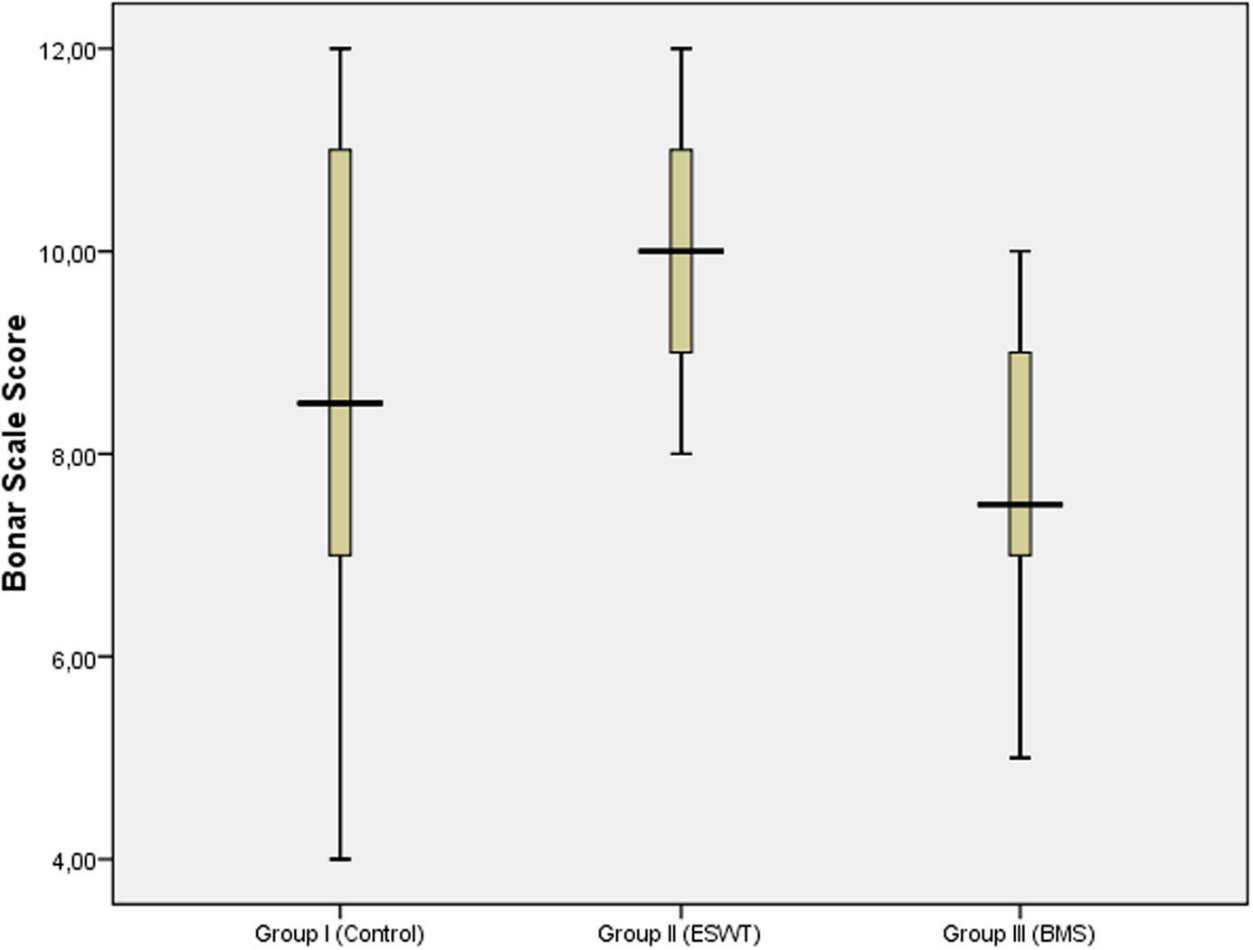
Comparison of Bonar scale scores among all groups at 4 and 8 weeks. The distribution of histopathological tendon scores, with the median line indicating the central tendency and whiskers representing the range. Lower Bonar scores indicate better tendon healing quality. BMS, bone marrow stimulation; ESWT, extracorporeal shockwave therapy.

At the 4‐week mark, Group I ground substance structure most closely resembled that of normal tendon in comparison to the other groups (*p* < 0.026) (Table 1). There were no statistically significant differences detected in collagen organization across the groups; a notable deterioration in tenocyte morphology was identified in Group II between the 4‐ and 8‐week time points (Figure 3). Group II and Group III exhibited more organized and tendon‐like vascular structures than Group I (*p* > 0.05). Group III exhibited the lowest overall Bonar scores, and only vascular patterns displayed the most normal vascular architecture (*p* < 0.014) (Table 2, Figure 4). In our study, to ensure consistency, evaluations were performed blindly and at two separate time points. Interobserver reliability for Bonar scale score was good (ICC: 0.772; 95% CI: 0.509–0.893). Two‐group comparison (ESWT vs. BMS) of Bonar scores with equal‐variance assumptions checked. The effect size was Hedges' *g* = 1.294 (95% CI: 0.33–2.26), with *p* = 0.008 and post hoc power = 0.756. Comparing all groups (Control, ESWT, BMS) at Week 8, the observed effect size was Cohen's *f* = 0.654, with *p* = 0.091 and post hoc power = 0.465.

**Table 1:** Comparison of ground substance and tenocyte scores according to sacrifice times.

		Group I (*n* = 10)		Group II (*n* = 9)		Group III (*n* = 10)	
		Sacrificed at 4 weeks (*n* = 5)	Sacrificed at 8 weeks (*n* = 5)	Sacrificed at 4 weeks (*n* = 5)	Sacrificed at 8 weeks (*n* = 4)	Sacrificed at 4 weeks (*n* = 5)	Sacrificed at 8 weeks (*n* = 5)
Ground Substance Score	0	0	0	0	0	0	0
	1	2	1	0	0	3	2
	2	0	4	4	2	0	1
	3	3	0	1	2	2	2
*p*		0.026^a^		0.343		0.549	
Tenocyte Score	0	0	0	0	0	0	0
	1	1	1	0	0	1	2
	2	3	1	4	0	3	2
	3	1	3	1	4	1	1
*p*		0.368		0.016^b^		0.766	

^a^ Ground substance structure most closely resembled that of normal tendon in comparison to the other groups.

^b^ A notable deterioration in tenocyte morphology was identified between the 4‐ and 8‐week time points.

**Table 2:** Comparison of Bonar scale score according to groups.

		Group I (*n* = 10)	Group II (*n* = 9)	Group III (*n* = 10)	*p*
Tenocyte Score	0	0	0	0	0.373
	1	2	0	3	
	2	4	4	5	
	3	4	5	2	
Ground Substance Score	0	0	0	0	0.075
	1	3	0	5	
	2	4	6	1	
	3	3	3	4	
Collagen Score	0	0	0	0	0.459
	1	1	0	1	
	2	3	3	6	
	3	6	6	3	
Vascularity Score	0	0	0	0	0.014^a^
	1	2	0	6	
	2	1	7	2	
	3	7	2	2	

^a^ Group III exhibited the lowest overall Bonar scores, and only vascular patterns demonstrated displaying the most normal vascular architecture.

### Immunohistochemical evaluation

**Figure 5:**
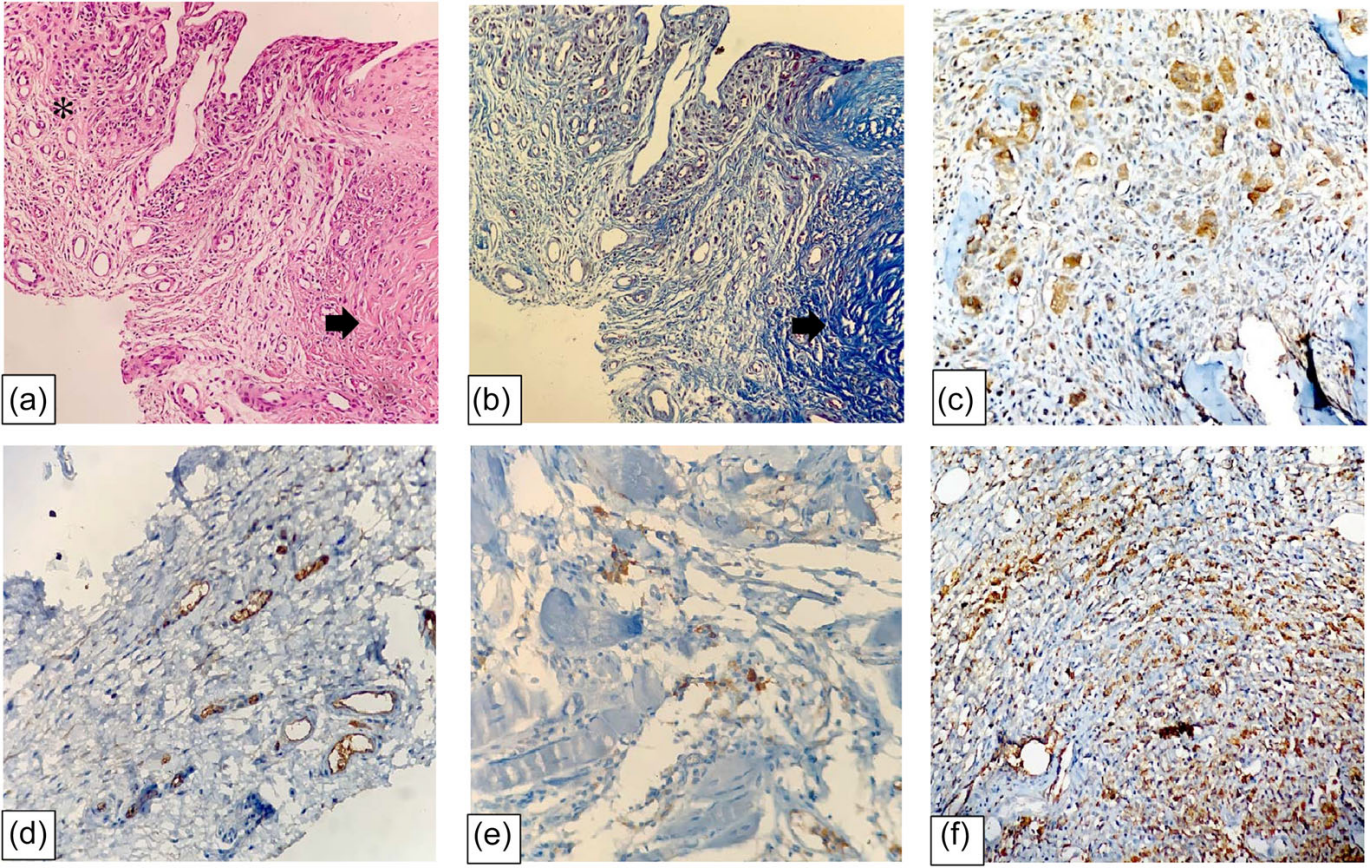
Representative images of immunohistochemical staining and histopathological assessment from study specimens. (a) At Week 4, Group II, increased neovascularization (asterisk) is observed between collagen fibre bundles (arrow) within the tendon tissue (H&E staining, ×100 magnification). (b) At Week 4, Group II, collagen fibres stained blue and vascular structures were clearly distinguishable (Masson's trichrome staining, ×100 magnification). (c) Strong cytoplasmic immunopositivity for BMP‐7 in multinucleated cell formations, Group III, staining observed in cell clusters at the tendon–bone interface at Week 8 (immunoperoxidase staining, ×400 magnification). (d) Positive immunopositivity for CD34 was observed in vascular endothelial cells within the tendon region at Week 4, Group II (immunoperoxidase staining, ×200 magnification). (e) Membranous TGF‐β1 staining in the tendon–muscle junction at Week 4, Group I, showing sparse immunopositivity (immunoperoxidase staining, ×400 magnification). (f) Cytoplasmic immunopositivity for BMP‐2 at the tendon–bone interface at Week 8, Group III (immunoperoxidase staining, ×400 magnification). BMP‐2, bone morphogenetic protein‐2; BMP‐7, bone morphogenetic protein‐7; TGF‐β1, transforming growth factor‐β1.

No significant differences were observed among the groups in TGF‐β1. Nevertheless, the expression of TGF‐β1 remained consistent in Group I, in contrast to the augmented groups. Immunoreactivity for CD34, a marker of vascular endothelial activity, was most evident in Group II (*p* > 0.05). Group III exhibited higher expression levels of BMP‐2 and BMP‐7 compared to the other groups (*p* > 0.05) (Figure 5).

## DISCUSSION

Group III exhibited the most comparable vascularity to that of normal tendon tissue (*p* < 0.014). Vascularity exhibits a dual role in the process of tendon healing. Although angiogenesis is necessary for early healing, excessive or prolonged vascularity has been linked to tendinopathy and pain [24]. In a similar vein, cadaveric studies in human subjects revealed that non‐injured tendons exhibited minimal vascularity, while surgically repaired tendons demonstrated increased vascularity [20]. As vascular integrity declined, clusters of capillarization became more prominent, suggesting a compensatory but potentially disorganized angiogenic response.

The utilization of techniques such as microfracture and multiple‐channelling of the greater tuberosity has yielded encouraging preliminary results [16]. However, according to a recent systematic review, BMS reduced retear rates in large or MRCT but did not result in clinically meaningful improvements in patient outcomes. This raises reasonable reservations regarding its routine use [2, 35].

The hypothesis for improved tendon‐to‐bone repair after BMS application may involve augmented secretion of healing agents, such as growth factors, fibroblasts and MSCs from the humeral head's BM at the site of repair. Kida et al. demonstrated in a rat model that BM‐derived cells migrated from drill holes to the repaired tendon, leading to improved histological organization and mechanical strength [16]. Jo et al. demonstrated that cells derived from the proximal humerus exhibited characteristics similar to those of endogenous MSCs following this methodology [15]. In our study, BMS was associated with increased vascularity and altered expression of BMP‐2, BMP‐7 at the tendon–bone interface, findings that are compatible with a pro‐regenerative microenvironment. These results are consistent with BM‐derived cells and their cytokine profile contribute to fibrocartilage formation and improved enthesis remodelling in rotator cuff repair [39].

ESWT, evaluated in this study as a non‐biological intervention, has been widely used in musculoskeletal conditions and has shown potential in enhancing tendon‐to‐bone healing. Its biological effects are known to be dose‐dependent, with in vitro and in vivo studies demonstrating variable outcomes based on energy levels and application parameters. While ESWT has been associated with reduced matrix metalloproteinase and cytokine levels, increased tenocyte proliferation and fibrocartilaginous regeneration at the enthesis, there is currently no consensus regarding optimal energy settings. The definitions of low, medium and high energy levels exhibit variability across studies. For instance, Speed et al. defined low energy as less than 0.10 mJ/mm^2^, whereas Han et al. reported a dose‐dependent decrease in cell viability at 0.17 mJ/mm^2^ [12, 31]. Recent studies found ESWT improved rotator cuff repair in rats; the observed neovascularization at the musculo‐tendinous transition zone is hypothesized to be the key factor [7]. Feichtinger et al. provide strong evidence that ESWT significantly improves biomechanical outcomes in surgically repaired chronic RCTs in rodents, with particular functional benefits observed in the repetitive ESWT group [8]. Accordingly, we planned direct comparison of the two techniques, each reported to be advantageous versus no adjunct, in a chronic massive rotator cuff repair model to determine whether either confers superiority.

In histomorphology evaluation, Group III demonstrated lower average Bonar scores compared to the other groups (*p* = 0.104), indicating a tendon structure more closely resembling normal tissue, such as enhanced tenocyte morphology and improved the ground substance structure. However, the statistically significant enhancement was observed exclusively in vascularity, thereby supporting the potential of BMS to promote neovascularization and facilitate tendon healing over time. However, across all time points, no statistically significant differences in Bonar scores were observed among the groups (*p* = 0.104).

In Group I, a gradual improvement in the ground substance was observed over time (*p* < 0.026). This suggests that Group I exhibited a delayed normalization of the tendon–bone interface without augmentation. However, despite these trends, no statistically significant differences were found among the groups in terms of ground substance scores at 8 weeks.

In Group II, in terms of tenocyte morphology worsened over time; animals at 8 weeks showed greater abnormalities compared with 4 weeks (*p* < 0.016). However, this alteration over time, rather than an improvement in the quality of the tenocyte morphology. No significant deterioration in tenocyte structure was observed at first‐time application of ESWT; however, a second identical application at 6 weeks coincided with a significant decline in tenocyte morphology by 8 weeks. In our model, ESWT likely produced anti‐inflammatory neuroimmune modulation while exceeding the tissue‐specific therapeutic window with the second exposure, leading to tenocyte/matrix injury despite reduced inflammatory infiltration. This pattern is consistent with the biphasic dose–response reported in tendon studies and with literature on M2‐skewing/neuropeptide modulation at lower loads [32]. This finding suggests that even within the low energy range, cumulative exposure may exceed the threshold for cellular tolerance, potentially resulting in structural degeneration. The precise ESWT dosage and duration that was administered remain to be determined.

In immunohistochemical evaluation, the expression of CD34 was found to be most prominent in Group II (ESWT), suggesting a directional increase in microvascular activity; this difference did not reach statistical significance (*p* > 0.162); nonetheless, the observed trend parallels previous reports of ESWT‐induced angiogenesis and underscores the need for precise dosing regimens and further studies to confirm these findings.

The augmentation groups exhibited TGF‐β1 expression consistent with resolution of the inflammatory phase, while several animals in the control group still exhibited elevated levels, suggesting ongoing inflammation. This observation is consistent with the established function of TGF‐β1 as the initial inflammatory phase of tendon healing [13]. It has been posited that persistent expression at subsequent time points may signify delayed progression toward the remodelling phase and inferior tissue maturation, particularly in the absence of biological stimulation. These findings support the potential of both ESWT and BMS to accelerate the transition out of the inflammatory phase, potentially contributing to improved tendon‐to‐bone healing in chronic RC tears.

BMP‐2 and BMP‐7 cytokine expression in Group III exhibited elevated at the 4‐week mark in comparison to the other groups. Cytokine levels exhibited a tendency to decrease over time, with the lowest expression levels being detected in Group I at 8 weeks. However, across all time points, the expression of BMP‐2 and BMP‐7 was most frequently observed in Group III throughout the study period; no statistically significant differences were observed.

### Limitations

This study is not without its limitations. It was conducted using a rat model, which, like other animal models, differs anatomically and functionally from the human shoulder. However, the rat supraspinatus tendon passes beneath the acromion during movement, resembling overhead activities in humans [30]. The presence of an acromial arch serves to further substantiate its relevance. Although primate models offer a closer resemblance to human shoulder anatomy, they are less practical due to their high cost, limited accessibility and ethical considerations. Consequently, despite anatomical variations, the rat persists in its role as a recognized and extensively utilized model for investigating rotator cuff pathologies [19, 26].

In histomorphology and immunohistochemical evaluations, despite its nature as a semi‐quantitative method, the Bonar scale has been demonstrated to possess a degree of reliability in prior studies [6, 21]. Maffulli et al. found that Movin's and Bonar's scores have a high correlation and assess similar characteristics and variables of tendon abnormalities, and Bonar scale score intraclass correlation was 0.921 (95% CI: 0.790–0.963) [21].

The comparison across groups was restricted to the tendon–bone junction; fatty degeneration was not evaluated and is therefore not reported in the study. Fatty degeneration is a significant clinical problem in humans following RCTs. However, it is noted that substantial fatty degeneration does not reliably occur in rodent models, including rats, after a supraspinatus tear unless both the muscle and its nerve (suprascapular nerve) are injured [29, 33].

In humans, chronic RCTs generally manifest over protracted periods as a consequence of internal and/or external factors. In this study, a chronic tear was simulated by surgically detaching the supraspinatus and infraspinatus tendons and delaying repair for a defined period. While this method does not fully replicate the chronicity observed in humans, previous studies have validated its use in rat models with histological analysis [22].

The chronic tear model was maintained for a period of four weeks, which is shorter than the chronic progression typically observed in humans (6–12 months) [11]. However, given the elevated metabolic rate and augmented healing capacity exhibited by rats, this time frame is deemed adequate to induce chronic tissue alterations [19, 26, 36]. One study posited that a period of one year in human aging is equivalent to approximately 28.7 days in rats, a conclusion derived from comparative metabolic rates [1].

Finally, in accordance with ethical principles, the number of animals per group was maintained at the minimum level required for statistical analysis. While this approach reduces the utilization of animals, it may also diminish the statistical power, particularly in semi‐quantitative assessments. When all time points were analysed together, the ESWT group had a worse histological profile than the BMS group (post hoc power = 0.756). However, statistical precision was limited at most time points, particularly in secondary analyses, resulting in a high risk of Type II error. Consequently, several results displaying directional trends toward significance did not reach statistical significance. As the study is histopathological and immunohistochemical without biomechanical testing, clinical inference is indirect. Subsequent studies with larger sample sizes may offer more robust results.

## CONCLUSIONS

Both biological augmentations using the BMS technique and ESWT demonstrated beneficial effects on tendon‐to‐bone healing in a rat model of chronic MRCT. Deterioration of tenocyte morphology over time in the ESWT group underscores the necessity of optimizing energy parameters. Significant improvements were only primarily observed in vascularity in Bonar score in Group III. Considering the inherent limitations of small animal models and fixed observation periods, further studies with larger sample sizes are imperative to validate and expand upon these findings.

## AUTHOR CONTRIBUTIONS

Utku Demirtaş participated in developing the scientific study plan and hypothesis. He performed the animal interventions and wrote the article. Mert Özcan developed the scientific study plan and hypothesis. He observed the animal interventions and reviewed the written article. Elif Mercan Demirtaş and Fulya Öz Puyan were responsible for the histopathological and immunohistochemical evaluation sections of the study.

## CONFLICT OF INTEREST STATEMENT

The authors declare no conflicts of interest.

## ETHICS STATEMENT

This study received approval from Trakya University, Local Ethics Committee of Animal Experiments, Approval Number: 2019.12.01.

## Data Availability

The image data supporting the findings of this study are openly available in Zenodo at https://doi.org/10.5281/zenodo.17833688. The remaining data that support the findings of this study are available from the corresponding author upon reasonable request.
